# How Does Joint Media Engagement Affect the Development of Executive Functions in 5- to-7 year-old Children?

**DOI:** 10.11621/pir.2023.0407

**Published:** 2023-12-01

**Authors:** Daria A. Bukhalenkova, Elena A. Chichinina, Olga V. Almazova

**Affiliations:** a Lomonosov Moscow State University, Russia

**Keywords:** preschool age, joint media engagement, screen time, executive functions (EF), working memory, inhibition, cognitive flexibility, digital devices (DD)

## Abstract

**Background:**

Executive functions are actively developing in children of preschool age. Executive functions’ development is also influenced by the way children are using digital devices. Joint media engagement is one of the parameters of digital device usage that has been poorly studied so far, although this is of great importance from the point of view of cultural-historical psychology.

**Objective:**

Our research aimed to explore the association between young children’s development of executive functions over a year, and their joint media engagement with parents and siblings in preschool children.

**Design:**

Four hundred ninety (490) typically developing children (52% of them were boys) participated in the study. It was a longitudinal study: during the first stage, the children were 5-6 years old; the second stage followed one year later. The NEPSY-II subtests (Inhibition, Statue, Memory for Designs, Sentences Repetition) and the Dimensional Change Card Sort were used to assess executive functions. A questionnaire for mothers was used to get information about the children’s joint media engagement and screen time.

**Results:**

Children who watched video content and played video games together with their siblings developed more inhibitory control over the year than those children who did it alone. Co-viewing of video content with parents was associated with a decrease in cognitive flexibility over the year, as opposed to watching it alone.

**Conclusion:**

The obtained data allows us to conclude that joint media engagement is important for executive functions development, and that there are optimal formats of joint media engagement. Based on the limitations of this study, recommendations for future research were suggested.

## Introduction

Preschool children are actively developing their executive functions (EF) (Blair & Raven, 2015; Garon et al., 2008). EF development in the preschool period predicts successful adjustment to school and schooling, as well as life achievements, health, well-being, and quality of life in adulthood (Banshchikova et al., 2023; Robson et al., 2020; Scionti et al., 2020; Stichter et al., 2016; Vets, 2023). EF development at preschool age is influenced by the social situation of a child’s development (Vygotsky, 1984), and the children’s usage of digital devices is a part of that context (Kurilenko et al., 2022; Soldatova, 2018). The term “digital devices” (DD) refers to electronic devices that have a screen — possibly to be used interactively — and potential access to the Internet, namely: TVs, smartphones, computers, and tablets.

Modern preschoolers use DD for about three hours a day (Konca, 2022; Rideout & Robb, 2020), and now the connection between the features of the DD use and EF development is being actively studied. A large number of studies have shown that screen time is inversely related to the development of all EF components in preschool children, but this applies primarily to those children who exceed the daily norm for screen time (Corkin et al., 2021; Jusienė et al., 2020; Linebarger et al., 2014; McNeill et al., 2019; Nichols et al., 2022).

When assessing the potential impact of DD use on EF development, it is important to study certain aspects of digital leisure such as the degree of its interactivity; however, this aspect has so far been poorly researched. Based on the degree of interactivity experienced in digital leisure, passive and active screen time can be distinguished (Bukhalenkova et al., 2021). Passive screen time is watching video content; active screen time involves cognitive and/or physical engagement in the process of using a digital device: for example, playing video games and using mobile applications.

Another little-described aspect of preschoolers’ digital experience is their joint media engagement with parents and siblings (Stevens & Penuel, 2010). Recent studies of joint media engagement have so far not dealt with the aspect of EF development (Dore & Zimmermann, 2020; Stevens & Penuel, 2010). Joint media engagement (shared DD use) includes watching video content without communicating, watching content with discussion, and joint video games, as well as the use of mobile applications (Dore & Zimmermann, 2020): that is, both passive and active screen time.

Thus, studying the joint passive and active screen time of preschoolers with their parents and siblings is important, since it can help determine the uses of DD which are most favorable for EF development (Belova & Shumakova, 2022). In this regard, the purpose of our research was to study the relationship between the development of the main components of EF in preschoolers over a year’s time, and with whom children spent their passive and active screen time — with parents, siblings, or independently.

### Executive functions development in preschool age

Our research focused specifically on studying the influence of how DD were used on EF development, since EF are an indicator of the mastery of higher mental functions. EF are a group of cognitive skills that enable goal-directed problem-solving and adaptation to new situations (Diamond, 2013; Friedman & Miyake, 2017). A. Miyake’s conceptual framework identifies the following components of EF: 1) working memory (verbal and visual) — this is the ability to retain information and use it to solve current problems; 2) cognitive flexibility — the ability to switch between tasks, rules, incentives, etc.; and 3) inhibitory control — inhibition of impulsive reactions and a dominant response in favor of what is required by the context (Diamond, 2013; Miyake et al., 2000).

EF development at preschool age depends on a large number of factors. First of all, it is mediated by various parameters of the child’s neurological development and his/her temperamental characteristics (Olness et al., 2009; Short et al., 2019). In addition, a sufficient quantity and quality of sleep (Kahn et al., 2021), as well as physical activity (Bai et al., 2020), are important. Second, EF development is affected by family lifestyle and parenting strategy, as well as the level of the parents’ education and family income (Hackman et al., 2015; Hughes & Devine, 2019). Also, the quality of parent-child relationships is significant for EF development. A child’s EF level is positively associated with the following characteristics of parental behavior: warmth, responsiveness, support, and willingness to join in the child’s activities, and provide him/her with independence (Valcan et al., 2018).

Third, EF development is favored by role play (Yogman et al., 2018; Veraksa et al., 2020b; Veraksa A. et al., 2022), which is the leading form of play at preschool age (Vygotsky, 2012). Th rough play, preschoolers develop the ability to follow rules, develop problem-solving skills, and master the main EF components (Yogman et al., 2018). Finally, as mentioned above, EF development at preschool age can be influenced by the child’s DD use, in particular parameters of use such as joint media engagement. The study of this particular parameter is of specific interest because it is a factor that parents can influence relatively easily, thereby promoting the development of a preschooler’s EF (Wannapaschaiyong et al., 2023).

### EF and joint media engagement of preschoolers and their parents

Joint media engagement of children and their parents may vary depending on the parental digital mediation strategy (Ewin et al., 2020; Kalabina & Progackaya, 2022). Two main parameters of parental digital mediation are parental support and parental control (Rudnova et al., 2023). Depending on the intensity of these parameters, the relationship between the child’s EF development and the joint media engagement of preschoolers and their parents may differ. On the one hand, it can be assumed that the predominance of parental support during digital mediation (information and technical assistance when using DD, discussion and explanation of the content, and emotional participation of the parent in the child’s digital activity) can contribute to the development of all EF components. On the other hand, it can be assumed that the predominance of parental intrusiveness and negative control during digital mediation can lead to a slower increase in inhibitory control, because it interferes with children’s own initiative (Geeraerts et al., 2021; Feng et al., 2011). However, joint media engagement of children and their parents is usually a manifestation of parental support during digital mediation, and, accordingly, should be beneficial for EF development (Wannapaschaiyong et al., 2023).

There is “high-level” and “low-level” joint family media engagement (Koran et al., 2022). “Low-level” joint media engagement is passively watching videos or playing games together without communicating or training; even this, compared to independent DD use, improves children’s understanding of what they see on the screen (Dore & Zimmermann, 2020). After all, even if a parent passively watches the video next to the child, he/she can answer questions about what he/she saw and express his/her attitude towards it (Waters et al., 2016). In a study by Wannapaschaiyong et al. (2023) with the participation of 110 5-6 year-old children, it was demonstrated that, when watching video content together, all parents in one way or another initiated a discussion with the child of what they saw together.

However, of course, “high-level” — that is, active and thoughtful — joint media engagement has a greater impact on children’s EF development (Dore & Zimmermann, 2020; Strouse et al., 2013). If the parent and child regularly discuss content while watching it, the negative effects of violent content on the child are mitigated; conversely, the positive effects of educational content are enhanced (Waters et al., 2016). It is important to note that most video games and mobile applications are designed to involve only one person (Dore & Zimmermann, 2020). Therefore, often joint engagement does not imply full interaction between the child and the partner, but rather observation of the partner’s activities. That is, “high-level” joint media engagement often requires special efforts on the part of the parent. But it is the “high-level” joint media engagement that can contribute to the development of all components of EF, since it is a form of child-parent interaction.

Joint media engagement of children and parents or other significant adults may be associated with EF development for a number of reasons. First, parents can make decisions regarding the selection of educational video content and video games that are most suitable for the child (Dore & Zimmermann, 2020; Ewin et al., 2020). Parents can also protect their child from unwanted content and various risks on the Internet. It has been shown that the content of videos, as well as of video games, can be associated with EF development: for example, educational digital activities aimed at children can contribute to EF development, and vice versa; content that is not suitable for those of a young age and content, for example, containing scenes of violence, can negatively affect EF (Bukhalenkova et al., 2021). Second, parents or other significant adults can help the child critically comprehend the video content he/she sees and discuss with the child his/her impressions after the digital leisure (Dore & Zimmermann, 2020). That is, the joint media engagement of children and parents or other significant people is a form of live communication, and high-quality child-parent interaction which can contribute to EF development (Veraksa, 2014). Third, parents or other significant adults can monitor a child’s screen time, while excessive screen time is a risk factor for EF development (Swider-Cios et al., 2023; Veraksa N. et al., 2022).

While in general the joint media engagement of preschoolers with their parents rather favors EF development, it can be assumed that there are also ways of joint media engagement that do not contribute to that positive outcome. First, if parental control significantly predominates in digital mediation, then it is likely that joint media engagement will not develop the child’s EF (Geeraerts et al., 2021; Feng et al., 2011). After all, when an adult overly controls the activities of a preschooler and does not give him/her the opportunity to take initiative, make decisions, or independently follow a plan, then the adult, as it were, takes on the role of the child’s EF. Second, there is a lack of scientifically proven evidence on how to find and choose high-quality educational apps (Aleksandraki & Zaranis, 2023; Belolutskaya et al., 2023; Papadakis et al., 2021; Pesha, 2022). A study by Papadakis et al. (2022) showed that parents prefer to download free apps for children. But such apps may include advertising that overstimulates a child’s attention. Also, joint media engagement may not contribute to EF development because parents do not have sufficient information on how to properly conduct digital mediation and how to talk about DD use (Papadakis et al., 2022). However, from the point of view of cultural-historical psychology, it is the child-parent interaction in the process of any activity that is important for EF development (Vygotsky, 2012). Based on this, the process of child-parent interaction itself during digital leisure is more important than choosing high-quality apps or video content. Thus, the impact of joint media engagement of preschoolers and their parents on the children’s EF development may be multidirectional, and it has so far been poorly studied.

### EF and joint media engagement of preschoolers and their siblings

No studies have been found on how the joint media engagement of preschoolers and siblings is related to EF development. So far, one can only make guesses about how joint media engagement of preschoolers and siblings affects EF development.

There are studies on how the presence of siblings and joint activities with them are generally connected to EF development. The results of these studies can also be partially applied to digital leisure. Thus, the presence of siblings in itself may be associated with a higher EF level (McAlister & Peterson, 2013; Rolan et al., 2018).

There are several reasons why the presence of a brother or sister may be associated with better EF development. First, the interaction of a preschooler with an older brother or sister is a context in which a preschooler can learn, and master new skills and cultural norms, thereby also developing EF (Vygotsky, 1978). Second, cooperative play and conflicts with siblings (whether younger or older) are safe contexts for the development of various social, emotional, and cognitive skills, including EF (McAlister & Peterson, 2013; Rolan et al., 2018). Having a sibling increases the frequency and variety of situations in which a child needs to compete and make compromises, potentially promoting the development of working memory, inhibitory control, and cognitive flexibility, as well as planning and strategic thinking skills (McAlister & Peterson, 2013). Third, parental upbringing strategies may differ depending on whether there is one child in the family or several. Thus, if there are siblings, parents can pay more attention to issues of discipline, structuring children’s leisure time, and introducing rules (Rolan et al., 2018), which is beneficial for EF development.

It is important to note that all these patterns apply primarily to siblings with a certain age difference. Thus, the interactions of an adolescent or adult sibling with a preschooler can resemble interactions between parents and a child, and create a social environment similar to that in which there is only one child (McAlister & Peterson, 2013). And if a preschooler has a much younger sibling, then interactions with him/her are limited and cannot fully contribute to EF development (McAlister & Peterson, 2013).

Based on the studies described above and the logic of EF development, we can assume that watching video content together with a sibling, as well as playing video games together, develops inhibitory control. This is because the child, in the process of sharing a DD with a sibling, needs to wait his or her turn, and not interfere with the partner. Playing multiplayer video games with a sibling is likely to develop working memory, because it is necessary to retain agreements about the shared actions and wishes of the partner, as well as the partner’s game actions themselves. Also, playing video games together with a sibling can help to develop cognitive flexibility because the child needs to constantly switch between two processes: communication with a sibling and the video game itself. Empirical research on this topic is required to test these assumptions.

### Our research

Despite the widespread use of DD by children, the connection between EF development and interaction with those with whom the child usually uses DD (with siblings, with parents, alone) has not been sufficiently studied. Some papers on joint media engagement of children and their parents are theoretical in nature, and there are not enough empirical studies (Dore & Zimmermann, 2020). We found no studies on how joint media engagement of children and their siblings affects EF development. But this topic is very relevant, as the senior preschool age (when interaction with other children first begins) is important for cognitive and emotional-personal development, particularly EF development (Lisina, 2009).

To fill the gap in existing knowledge, we set out to study the relationship between the rate of development of EF in preschool children over one year (from 5 to 6 years old), and the characteristics of joint media engagement of children and their parents and siblings. The following research questions were formulated:

1. How is the joint media engagement of preschoolers and their parents related to the development of various components of EF over one year?
2. How is the joint media engagement of preschoolers and their siblings related to the development of EF’s various components over one year?

At the same time, the factor of screen time was taken into account as a parameter potentially influencing EF development (Corkin et al., 2021).

## Method

### Participants

Four hundred ninety (490) children (52% of them were boys) from municipal kindergartens in three regions of Russia took part in our longitudinal study: 35.6% of children were from Kazan, 32.5% from Moscow, and 31.9% from the Republic of Sakha (Yakutia). Seventy-four percent of the mothers had a higher education; 78% of the families had an average level income.; and 67% of the children had one or more siblings.

During the first phase of the study, the average age of the children was 5 years and 5 months (M = 65.14; SD = 5.04 months). The children were pupils in the senior groups of the kindergarten. The second phase was carried out a year later, when the children were attending the kindergarten’s preparatory groups, which is the last educational stage in the kindergarten, before the children go to school.

### Materials

**To study the main EF components** (working memory, inhibition, and cognitive flexibility), we used a set of tasks which had been previously tested on a Russian sample (Veraksa et al., 2020a).

**For verbal working memory** assessment**,** the NEPSY-II subtest “Sentence Repetition” (Korkman et al., 2007) was used. The stimuli consisted of 17 sentences of increasing length and complexity. Each sentence was read out loud to a child, and then he/she was asked to repeat it immediately. Each correctly repeated sentence was scored 2 points. If the child made one or two mistakes, the response was scored 1 point; if there were three and more errors during the repetition, the sentence was scored 0 points. The exercise stopped when the child received 0 points three times in a row. Accuracy scores were also calculated (max 34 points).

**For visual working memory** assessment, the Memory for Designs subtest of the NEPSY-II (Korkman et al., 2007) was applied. This technique included four tasks; in each, the child was presented with a grid (a field 4 by 4 with 16 cells) where several (from 4 to 8) color pictures were located in different cells. The child was shown a picture for 10 seconds, and then the picture was taken out of sight. The child then had to select the patterns from a set of pictures and place them in a grid in the same place they were shown earlier. The child had to complete four tasks. For each task, points were scored on four indicators: 1) the Content Score evaluated the child’s ability to remember image details (to choose those pictures that were in the example, not distractors); 2) the Spatial Score evaluated the child’s ability to remember the location of objects (to place the cards in the correct cells on the grid); 3) the Bonus Score reflected the child’s ability to correctly reproduce the entire visual image (put the correct cards on the right places on the grid); and 4) the Total Score was the sum of the three previous indicators (max 120 points).

**For cognitive inhibition** assessment, the Inhibition subtest of the NEPSY-II (Korkman et al., 2007) was used. The subtest included two series of black and white pictures: a series of figures (circles and squares) and a series of differently directed (up and down) arrows. Two tasks were required with each series of pictures: 1) the Naming task (a child had to name the figures that she/he saw as quickly as possible) and 2) the Inhibition task (a child had to say the opposite of what he/she saw: for example, if she/he saw a circle, she/he had to say “square”). Three metrics were analyzed in each task: 1) the number of uncorrected errors which occurred when the child did not correct the mistakes made; 2) the number of self-corrected errors which occurred when the child at first gave the wrong answer, but then corrected himself/herself; and 3) the time that it took the child to name all the figures. These three scores were then converted into a combined score using special tables from the NEPSY-II manual (from 1 to 20 points).

**For physical inhibition** assessment, the Statue subtest of the NEPSY-II (Korkman et al., 2007) was used. In this technique, the child had to stand motionless with closed eyes in a certain position for 75 seconds, without being distracted by external sound stimuli (tapping, coughing, the sound of a pen falling on the floor, etc.). For each 5-second interval three types of mistakes were recorded (*i.e.*, movements, the opening of the eyes, vocalizations); the child received two points if she/he made no mistakes during the 5-second interval; one point if child made one type of mistake; and 0 points if child made two or more types of errors (max 30 points).

**For cognitive flexibility** assessment, the Dimensional Change Card Sort task (Zelazo, 2006) was used. The children were required to sort a series of cards with pictures of red rabbits and blue boats following different rules. In the first task, the child sorts six cards by color (red ones are put in one direction, blue ones in the other). In the second, six cards are sorted according to the shape (boats are put in one direction, hares in the other). In the third task a child has to arrange 12 cards based on the complex rule: if the card had a black frame, then he/she had to sort it by color, and if there was no frame, then he/she had to sort it by form. For each correctly placed card, a child received one point; at the end the total number of points was calculated (max 24 points).

**To study the characteristics of DD use by the children**, an online questionnaire was distributed among the mothers; it contained questions about socio-demographic factors (place of residence, age and gender of the child), and the family’s socio-economic characteristics (income level, level of education of the mother). The mothers also had to estimate in minutes how much, on average, on a typical day the child spends watching video content, and how much the child play with the help of DD (separately for weekdays and weekends). Then the average weekly passive and active screen time of the children was calculated.

The following questions were asked regarding joint media engagement of children and family members:

1. “With whom does the child usually watch cartoons, films, and videos on the Internet or on TV?” Parents were asked to choose one of the following options: “Alone,” “With a brother or sister,” “With adult family members,” or “Other.”
2. “With whom does the child usually play on electronic devices?” Parents were asked to choose one of the following options: “Alone,” “With a brother or sister,” “With adult family members,” or “Other.”

In cases where parents chose the answer “Other,” their answers were not further analyzed.

### Procedure

The study was carried out in two phases, one year apart. During the first phase, the EF of the children from senior kindergarten groups were evaluated. At the same time, their mothers filled out online questionnaires about the specifics of their children’s use of DD. During the second phase, a year later, when the children were in the preparatory kindergarten groups, their EF were re-evaluated.

During both phases, EF assessment was carried out individually with each child, in a quiet room familiar to children (in their kindergarten). The evaluations were carried out over two meetings, each lasting approximately 20 minutes. During the first meeting, the children performed the tasks aimed at assessing their cognitive flexibility and inhibitory control; during the second one, the tasks aimed at assessing working memory. The sequence of tasks was fixed in both phases of the study and was the same. The tasks were split into two meetings in order to avoid overwhelming the children during the evaluation. Informed consent was obtained from parents for the participation of their children in the study.

During the first phase of the study, the children’s mothers received a link to the questionnaire that they filled out via an email from municipal educational organizations or in a parent chat in the WhatsApp messenger. It took the mothers approximately 20 minutes to complete the online questionnaire.

## Results

### Descriptive statistics

According to the data obtained from the survey of the mothers (see *Table 1*), approximately half of the children watched cartoons, films, and videos with their siblings (53.7%), while a third of the sample watched them alone. Only 15.6% of the children watched content with their parents. In the case of active screen time (play on electronic devices) compared to passive screen time (video watching), a larger number of children played on their own (40.1%), while the percentage of those playing with siblings decreased slightly; those playing with their parents remained approximately the same. Therefore, not all the children who had siblings, usually used DD with them.

**Table 1:** Survey results of and active screen mothers about how long time and with whom their child usually spends passive

	Passive screen time (n=475)		Active screen time (n=379)	
	With whom does the child usually watch cartoons, films and videos on the Internet or on TV?	Average screen time per day (min)	With whom does the child usually play on electronic devices?	Average screen time per day (min)
Alone	30.7%	114	40.1%	75
With siblings	53.7%	105	45.9%	85
With parents	15.6%	98	14.0%	45

It is crucial to emphasize that many parents reported that their child did not play on a digital device at all. Therefore, the number of children about whom parents answered the question “with whom the child plays using DD” (n = 379) was much lower than the number of children about whom parents answered the question “with whom does the child watch video content” (n = 475).

According to the data obtained, the children showed improvement in most EF components over the year (see *Table 2*). At the same time, the EF level at both the first and second stage of the study corresponded to the previously obtained norms for these ages in a Russian sample (Veraksa et al., 2020a).

**Table 2:** Descriptive statistics of EF evaluation results in preschool children ages 5 and 6 years

	First phase (5-6 y.o.)		Second phase (6-7 y.o.)	
	Mean	Standard deviation	Mean	Standard deviation
Cognitive Inhibition	10.2	3.09	11.4	3.28
Physical Inhibition	25.0	5.03	27.1	4.49
Cognitive Flexibility	20.4	3.01	20.0	2.44
Visual Working Memory	74.2	17.8	92.1	19.8
Verbal Working Memory	17.0	3.83	19.5	4.33

Due to the fact that the distribution of changes in the development rate of EF components over the year (the difference between scores) was not normal (according to the Shapiro-Wilk test), the nonparametric Mann–Whitney U test was used for further analysis.

### EF development over a year and the joint media engagement of preschoolers and their parents

Since there were significantly fewer children who often watched video content with adults than children who often watched video content alone, sex- and age-matched children were randomly selected from the latter group so that the groups were equal in size. The groups were similarly equalized in terms of both passive and active screen time with siblings.

**Figure 1.**
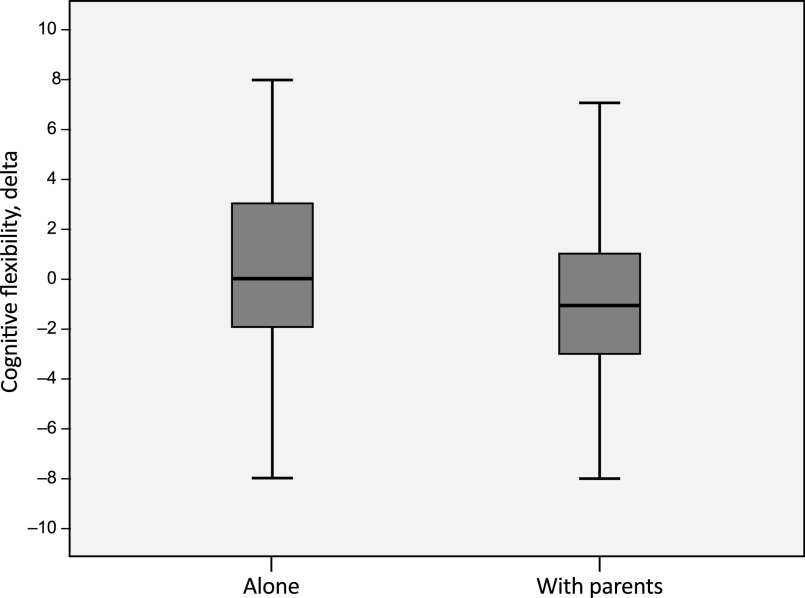
Differences in the cognitive flexibility development rate between children who watch video content alone and those who usually watch it with their parents

According to the data, the children who more often watched video content alone had a greater increase in cognitive flexibility skills over the year than those who more often watched video content with their parents (Mann-Whitney test U = 1842.500, p = .007). At the same time, the majority of children who watched content with their parents showed a decrease in the level of cognitive flexibility (see *Figure 1*). In addition, children who watched video content alone had statistically significant higher screen time that children who watched video content with their parents (Mann-Whitney test U = 4368.500, p = .029) (see *Table 1*).

There were no statistically significant differences in development of EF components between children who usually played alone on the DD and those who usually played with adults.

### EF development over a year and the joint media engagement of preschoolers and their siblings

**Figure 2.**
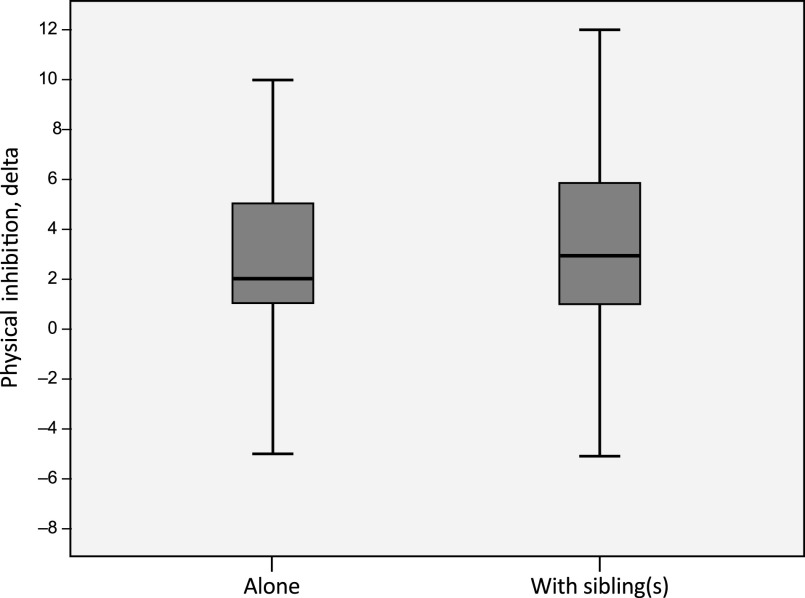
Differences in the physical inhibition development between children who watch video content alone and those children who watch it with siblings

**Figure 3.**
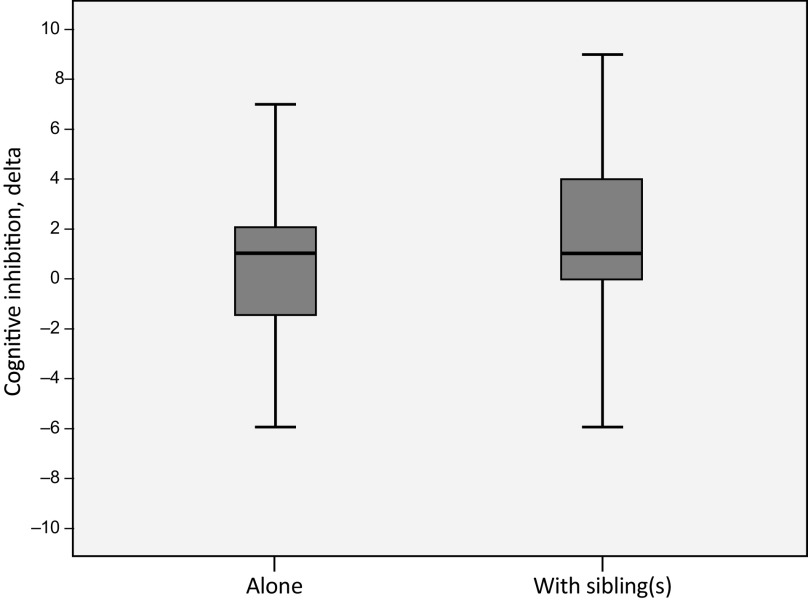
Differences in the cognitive inhibition development between children who play digital games alone and those who play with siblings

We found that physical inhibition increased over the year significantly more in those children who watched video content with siblings, compared to those children who more often watched it alone (Mann-Whitney test U = 5341.000, p = .011) (see *Figure 2*). Those children who often played on DD with their siblings showed a significantly higher increase in their inhibition over the year compared to those who more often played alone (Mann-Whitney test U = 5277.500, p = .018) (see *Figure 3*). The Mann-Whitney test showed that there were no statistically significant differences in either the passive or active screen time of the children who used the DD alone and with siblings (see *Table 1*).

## Discussion

The purpose of this longitudinal research was to study the relationship between the development of the main components of EF in preschool children over a year (from 5–6 to 6–7 years old) and the characteristics of joint active and passive screen time. We formulated a research question about how the development of EF component over a year was related to the characteristics of the joint media engagement.

As a result, it was discovered that many children who watched video content with their parents showed a deterioration in cognitive flexibility over the year, in contrast to those children who watched video content alone. There are several explanations for this result. First, it can be assumed that independent viewing of video content by preschoolers involves the child’s independent search and selection of cartoons or videos that interest him/her, which can contribute to his/her cognitive flexibility development. A child may use different strategies to find video content of interest. Also, when searching, the children have to pay attention to the different characteristics of video captions and splash images, which are important to consider in order to find what you are looking for. In the cases where children co-viewed video content with their parents, it can be assumed that the search and selection was carried out by the adult. Second, parents were also more likely to engage the types of media that they themselves used more often (Connell et al., 2015; Dore & Zimmermann, 2020). It can be assumed that in this case, the child’s cognitive flexibility might develop less actively, since the child was forced to follow the type of digital activity chosen by the adult and did not have sufficient experience in changing digital contexts to contribute to the cognitive flexibility development. Third, some parents, due to not having much digital competence, offered their child a small range of apps and video content, and did not know how to find appropriate educational content.

Finally, another reason may be that the joint media engagement was often “low-level” (Dore & Zimmermann, 2020), meaning that in the process, parents paid insufficient attention to discussing what they saw with the child and were not emotionally involved enough in this process (Ewin et al., 2020). In these cases, passive video content co-viewing did not become a source of live communication between a child and an adult, which could have strengthened child-parent relationships and contributed to EF development. It can be assumed that if a parent would ask the child questions about what he/she saw after co-viewing video content, — for example, about how the child understood what happened, about the reasons for the behavior of the characters and the consequences of their actions, about the child’s own thoughts and feelings— then this would contribute to both working memory training, as well as cognitive flexibility and other cognitive functions (Bukhalenkova et al., 2021; Ewin et al., 2020).

The screen time of those who usually watched video content on their own was higher than the screen time of those who usually watched with their parents. This result is inconsistent with existing evidence that more screen time is associated with lower rates of cognitive flexibility development (Bukhalenkova et al., 2021). It can be assumed that the difference of 16 minutes per day (114 minutes per day for those who watch alone, 98 minutes for those who watch with their parents) was not significant, and the features of sharing the control center played a larger role. However, it is possible that, unlike co-viewing time, the child’s independent viewing time of video content was not accurately known to parents, and they slightly overestimated it. However, in any case, this result needs to be rechecked and clarified.

At the same time, no significant differences were found in the development of all EF components between children who usually played with DD independently and children who usually played with their parents. The lack of differences may be due to the fact that most video games and mobile applications are designed to involve only one person (Dore & Zimmermann, 2020), so in such cases, joint media engagement often does not include full interaction between the child and the partner, but rather observation of the partner’s activities. Such a situation will be more likely to be typical for interaction with an adult, who can be either the main player (when a child watches one of the parents play) or, conversely, only a passive observer of the child’s play. In the case of senior preschoolers, the situation where an adult only watches his/ her child play is almost the equivalent of the situation of a solitary game; this may explain the lack of differences.

When playing video games together, children often act as two equal players. First, it is more interesting for them to compete with each other, and second, the two children both want to play on the DD and are often not ready to give in to each other, so they have to agree on the conditions of playing together, often changing roles (*e.g.*, they have to agree who is watching the game, and who is playing) (McAlister & Peterson, 2013; Rolan et al., 2018). In this regard, the differences we found in the inhibitory control development when the children were playing together with peers seem logical and natural. It was found that children who played video games and watched video content with siblings developed greater inhibitory control development over the course of a year than children who did so alone.

Interestingly, DD use with siblings to view video content (passive screen time) was associated with physical inhibitory control, which may be explained by the need to sit quietly and not interfere. On the other hand, DD use with siblings for games (active screen time) turned out to be associated with cognitive inhibitory control — that is, the ability to restrain impulsive reactions in the case of performing cognitive tasks. The situation of interaction with a sibling while playing undoubtedly contributes to cognitive inhibitory control training: an older sibling can teach a child new skills to cope with his/her behavior during play (Vygotsky, 1978), and a play situation with a younger sibling will contribute to training patience in a preschooler. In addition, in a situation of joint play with a younger sibling, the child can act as a teacher, which will also contribute to his/her self-regulation development, since he/she will need to take care not only of himself/herself, but also control the younger sibling.

Let us note that no statistically significant differences were found in either the passive or active screen time between children who used DD alone and with siblings, which allows us to conclude that the screen time factor did not influence the results obtained on the relationship between the EF development over the year and joint media engagement with siblings. Therefore, the data obtained allow us to conclude that at preschool age, joint media engagement with siblings has the most significant effect on inhibition development.

No significant differences were found in working memory and cognitive flexibility between children who usually used DD alone and those who used it with their siblings. To explain the absence of differences, further research on the nature of joint media engagement with siblings (how exactly the children interacted) is needed.

## Limitations

There were a number of significant limitations of this study. Let’s start with those associated with collecting information from the parents about the joint media engagement. First, in the questionnaire, parents could choose only one option, which limited their ability to reflect reality. For example, there could be children who used DD with approximately equal frequency on their own and with someone else. But the parents of such children were forced to choose only one answer. When using this questionnaire in the future, it is planned to make it possible to select several answers and indicate the frequency with which each option is practiced in the family. Second, parents, due to social desirability or limited awareness, may have given inaccurate answers to the questionnaire. For a more complete and objective picture, researchers can also interview the children themselves in the future. A third limitation was the lack of detail in the questions. For a deeper understanding of the topic of joint media engagement, it would be important to add more clarifying questions to the questionnaire about how exactly the joint media engagement occurred: *e.g.*, how the participants agreed among themselves, whether they discussed what they saw, or the experience gained. It also seems relevant to consider screen time. In addition, the age of the siblings was unknown, which is also a significant limitation of this work.

The next limitation of the study was that the survey on DD use was carried out only during the first phase. That is, the study was based on the assumption that the method of DD use did not change significantly over the year, while, in reality, the features of joint media engagement could have changed dramatically over the course of the year. For example, the family’s living conditions or the composition of the family could have changed. In the future, when conducting longitudinal studies, it is necessary to run a repeat survey at the final stage. Another limitation was the fact that the mere presence of a sibling may be associated with higher levels of EF (McAlister & Peterson, 2013; Rolan et al., 2018). So, the finding that joint media engagement with siblings is associated with higher level of inhibition development may actually be explained simply by having a sibling and interacting with him or her.

Another significant limitation of the work was the differences in screen time between children who watched video content on their own and those children who watched video content with their parents. In the future, it is necessary to compare the features of shared use of DD between groups of children with equal screen time.

Also, this research did not take into account many other factors that could affect EF development, such as the quantity and quality of the children’s sleep (Kahn et al., 2021) and their physical activity (Bai et al., 2020), the children’s attendance at various additional classes (Dolgikh et al., 2023), and features of the parent-child relationships (Valcan et al., 2018) and others. It is important to take these parameters into account in future research. It is also necessary for future research to consider parents’ education level, family income, and parental attitudes toward DD, as these features may influence how parents use DD with their children (Aleksandraki & Zaranis, 2023; Papadakis et al., 2022; Waters et al., 2016).

## Conclusion

The data obtained in this study complements and expands our knowledge about the impact of joint media engagement on EF development at preschool age. It has been demonstrated that 5- to 6-year-old children who played video games and watched video content with siblings experienced more intensive inhibitory control development over the year than those children who did it alone. At the same time, the children who watched video content with their parents showed a deterioration in cognitive flexibility over the year, unlike those children who watched such content alone.

This research shows the importance of organizing joint leisure time with a child using DD, especially if the child uses them alone all the time. Based on the results obtained and analysis of the limitations of this study, suggestions for future research can be formulated. Thus, the survey questions about DD use should be made more precise and detailed in order to have more information. At the same time, in addition to the questionnaire for parents, it is worth using other sources of information (surveying the children themselves).
